# A New Cytotoxic Sesquiterpene Quinone Produced by *Penicillium* sp. F00120 Isolated from a Deep Sea Sediment Sample

**DOI:** 10.3390/md10010106

**Published:** 2012-01-12

**Authors:** Xiuping Lin, Xuefeng Zhou, Fazuo Wang, Kaisheng Liu, Bin Yang, Xianwen Yang, Yan Peng, Juan Liu, Zhe Ren, Yonghong Liu

**Affiliations:** 1 Key Laboratory of Marine Bio-Resources Sustainable Utilization, Guangdong Key Laboratory of Marine Materia Medica, RNAM Center for Marine Microbiology, South China Sea Institute of Oceanology, Chinese Academy of Sciences, Guangzhou 510301, China; Email: xiupinglin@hotmail.com (X.L.); xfzhou@scsio.ac.cn (X.Z.); wangfazuo@scsio.ac.cn (F.W.); bingo525@163.com (B.Y.); xwyang@scsio.ac.cn (X.Y.); py00_2006@126.com (Y.P.); ljuan2010@qq.com (J.L.); 2 Biomedicine Research and Development Center of Jinan University, Guangzhou 510632, China; Email: liukaisheng1987@163.com (K.L.); rz62@163.com (Z.R.)

**Keywords:** *Penicillium* sp. F00120, sesquiterpene quinone, cytotoxic, deep sea sediment, penicilliumin A

## Abstract

A new fungal strain, displaying strong toxic activity against brine shrimp larvae, was isolated from a deep sea sediment sample collected at a depth of 1300 m. The strain, designated as F00120, was identified as a member of the genus *Penicillium* on the basis of morphology and ITS sequence analysis. One new sesquiterpene quinone, named penicilliumin A (**1**), along with two known compounds ergosterol (**2**) and ergosterol peroxide (**3**), were isolated and purified from the cultures of F00120 by silica gel column, Sephadex LH-20 column, and preparative thin layer chromatography. Their structures were elucidated by detailed nuclear magnetic resonance (NMR) and mass spectroscopic (MS) analysis as well as comparison with literature data. The new compound penicilliumin A inhibited *in vitro* proliferation of mouse melanoma (B16), human melanoma (A375), and human cervical carcinoma (Hela) cell lines moderately.

## 1. Introduction

In recent years, marine microbes have received growing attention as the sources for bioactive metabolites. As a result of adaptation to the cold, high-pressure, low-nutrition, no light and local high temperature, high salt and other extreme ocean environments, marine microbes have formed unique genetic backgrounds and metabolic pathways [1]. The rich diversity of new bioactive compounds produced by these organisms suggests their importance as potential sources of pharmaceutical leads [2,3,4,5]. In particular, marine fungi have shown promising potential to produce a broad variety of bioactive metabolites with novel structures, and so far have provided more than 1000 new natural products, including polyketides, alkaloids, terpenoids, peptides, prenylated polyketides, shikimate-derived metabolites, and lipids [6,7,8,9]. Of these new natural products, 16% were isolated from marine sediment-derived fungi. The deep sea is emerging as a new and interesting source of such microbes, however, only a handful of reports have described new metabolites from fungi derived from this habitat. Moreover, all were obtained from deep sea sediments [8]. Plenty of secondary metabolites from marine fungi have been demonstrated to show significant cytotoxicity against tumor cell lines [2,8].

In this work, we describe the fungal strain F00120, which was isolated from a deep sea sediment sample and exhibited strong toxic activity against brine shrimp larvae. The isolate was identified as a member of the genus *Penicillium* on the basis of taxonomic experiments. One new sesquiterpene quinone (**1**), along with two known sterols (**2**, **3**), were isolated and purified from this marine fungus. Their structures were determined by nuclear magnetic resonance (NMR) spectroscopy and mass spectrometry (MS) analysis as well as comparison with literature data. The new sesquiterpene quinone displayed moderate cytotoxic activity against mouse melanoma (B16), human melanoma (A375), and human cervical carcinoma (Hela) cells.

## 2. Results and Discussion

### 2.1. Characterization and Identification of Isolated Strain F00120

**Figure 1:**
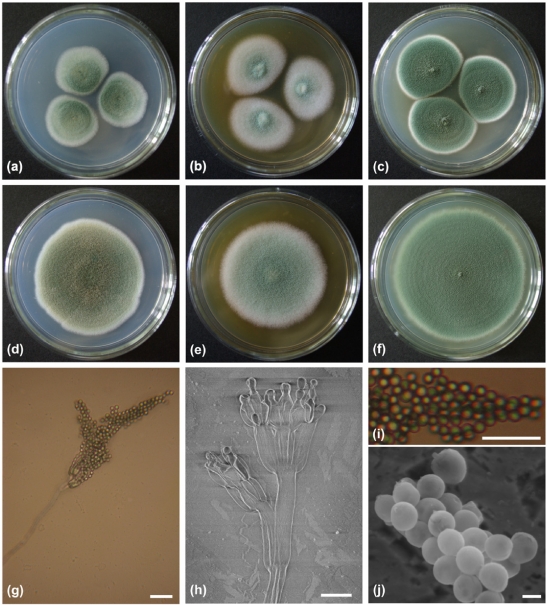
Colony appearance and micromorphology of *Penicillium* sp. F00120. (**a**–**c**) Colony appearance after 7 days at 25 °C (a, CA; b, SA; c, PDA); (**d**–**f**) Colony appearance after 14 days at 25 °C (d, CA; e, SA; f, PDA); (**g**) Conidiophore under light microscope; (**h**) Conidiophore under SEM; (**i**) Conidia under light microscope; (**j**) Conidia as seen using SEM. Bars: 10 µm (**g**–**i**) and 1 µm (**j**).

A fungal strain, F00120, which was isolated from a deep sea sediment sample collected in the northern South China Sea at a depth of 1300 m, displayed strong toxic activity against brine shrimp larvae. At a concentration of 20 mg/mL, the EtOAc extract of the fungal culture had a lethality rate of 95% to brine shrimp nauplii. On CA (Czapek’s agar) medium, after 7 days of growth at 25 °C, colonies were 39–41 mm in diameter, showed good sporulation, were mainly green at the centre and had a white periphery with a regular margin, whilst the reverse was dominated by a pale green color (Figure 1a). After 14 days of growth at 25 °C, colonies were 74–75 mm in diameter, displayed very good sporulation, were dominated by a deep yellowish green with a pale green/white edge and a light green/gray reverse (Figure 1d). On SA (Sabourand’s agar) medium, after 7 days of growth at 25 °C, colonies were 48–50 mm in diameter, showed good sporulation, had a very light green centre surrounded by a white edge with a regular margin and a reverse dominated by a pale orange-yellow color (Figure 1b). After 14 days of growth at 25 °C, colonies were 82–84 mm in diameter, presented good sporulation, were dominated by very light green centre, with a white edge, and a reverse that was light orange-yellow in color and, irregularly wrinkled (Figure 1e). On PDA (potato dextrose agar) medium, after 7 days of growth at 25 °C, colonies were 45–50 mm in diameter, displayed very good sporulation, were mainly deep green at the centre and surrounded by a white edge with a regular margin whilst the reverse was dominated by yellowish-gray, pale yellow exudate droplets (Figure 1c). After 14 days of growth at 25 °C, colonies were 84–86 mm in diameter, exhibited abundant sporulation, had a deep green centre surrounded by a very light yellow green edge, with a reverse dominated by a brilliant yellow green, notably the exudate droplets had disappeared (Figure 1f).

Scanning Electron Microscopy (SEM) revealed the penicilli were biverticillate (terverticillate), with smooth-walled to finely roughened stipes, 109.2–354.5 µm in length, and bore 2–3 smooth-walled to finely roughened rami measuring 10.0–27.5 (mean = 20) × 3.8–5.0 (mean = 4.3) µm. Metulae were smooth-walled and cylindrical measuring 7.5–13.3 (mean = 10.5) × 3.8–5.8 (mean = 4.8) µm. Phialides were found to be smooth-walled and flask-shaped, 6.3–17.5 (mean = 11.6) × 3.3–5.0 (mean = 4.0) µm, with rather long collula (1.5–2 µm). Conidia were green and globose to subglobose when mature, 2.2–3.2 (mean = 2.5) µm (Figure 1g–j).

**Figure 2:**
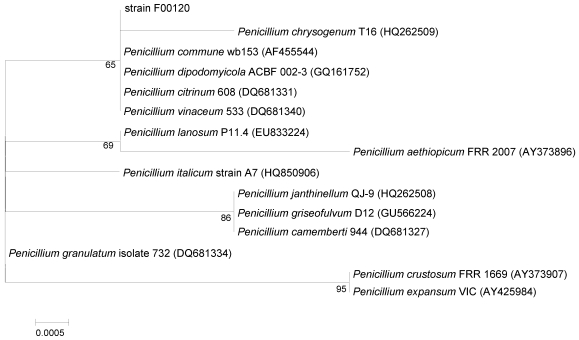
Neighbor-joining tree based on ITS1-5.8S-ITS2 sequences, showing phylogenetic relationship between strain F00120 and related *Penicillium* species. *Numbers at nodes* indicate bootstrap values from 1000 replicates. GenBank accession numbers are given in *parentheses*. Bar: 0.05% sequence divergence.

ITS1-5.8S-ITS2 sequence region (585 basepairs (bp), accession number JN380201) of strain F00120 was amplified by PCR and DNA sequencing showed it shared significant homology to several species of *Penicillium*, with sequence identities ranging from 100 to 99%. A phylogenetic tree was constructed, using the neighbor-joining method based on similarity of a 584-bp consensus length of ITS1-5.8S-ITS2 sequence (Figure 2). It showed that strain F00120 formed a monophyletic group with *Penillium chrysogenum*, *Penillium commune*, *Penillium dipodomyicola*, *Penicillium citrinum*, and *Penicillium vinaceum*, and that its closest neighbor was *P. chrysogenum* T16 (HQ262509). On the basis of its morphological property and ITS sequence, the fungus strain F00120 clearly belongs to the genus *Penicillium*, and was designated as *Penicillium* sp. F00120.

### 2.2. Structure Elucidation

**Figure 3:**
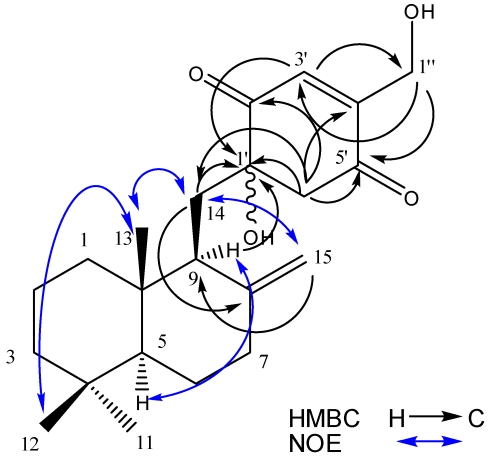
Key NOESY and HMBC correlations of penicilliumin A (**1**).

**Figure 4:**
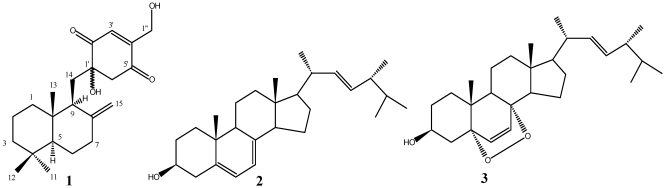
Structures of compound **1**–**3** from *Penicillium* sp. F00120.

Penicilliumin A was obtained as white crystalline solid, [α]_D_^20^: −0.008 (*c* 0.85, CHCl_3_). The IR spectrum possessed absorptions for hydroxyl groups (3436 cm^−1^) and carbonyl groups (1683 cm^−1^). It was analyzed for C_22_H_32_O_4_ by a combination of HREIMS (*m/z* 360.2296 [M]^+^, calcd 360.2295), ^13^C NMR and DEPT spectra indicating 7 degrees of unsaturation. The ^1^H NMR spectrum exhibited three methyl signals on quaternary carbons at δ_H_ 0.58, 0.76, and 0.86 (each 3H, s), a pair of hydroxylmethylene protons at *δ*_H_ 4.44 (d, *J* = 1.7 Hz) and 4.54 (d, *J* = 1.7 Hz), and three olefinic protons at *δ*_H_ 4.26 (br s), 4.75 (br s), and 6.82 (s). The ^13^C NMR and DEPT spectra showed signals representing three methyls (*δ*_C_ 15.0, 21.5, 33.6, each CH_3_), one oxygenated methylene carbon (*δ*_C_ 59.7 CH_2_) and one oxygenated tertiary carbon (*δ*_C_ 77.0 C), four olefinic carbons (*δ*_C_ 107.0 CH_2_, 134.3 CH, 148.9 C, 150.8 C), and two ketone carbonyl carbons (*δ*_C_ 201.1 C, 196.5 C). The ^1^H and ^13^C NMR spectroscopic data of this compound (Table 1) resembled those of tauranin, containing the same sesquiterpene quinone carbon skeleton [10]. Tandem EI-MS produced a fragmentation ion at *m/z* 205 [C_15_H_25_] which confirmed the sesquiterpene moiety of penicilliumin A was the same as that in tauranin. The major differences were the absence of one double bond and position of one hydroxyl group in quinone moiety of penicilliumin A. In the HMBC spectrum (Figure 3), the protons of H-6′ (*δ*_H_ 2.97, d, *J* = 1.6 Hz; 3.12, d, *J* = 1.6 Hz) showed correlations with C-1′ (*δ*_C_ 77.0), C-2′ (*δ*_C_ 201.1), C-4′ (*δ*_C_ 150.8), and C-5′ (*δ*_C_ 196.5), supporting the absence of the double bond at C-1′and C-6′. Other HMBC correlations of the signals H-9 (*δ*_H_ 1.79), H-14 (*δ*_H_ 1.80, 1.89), and H-3′ (*δ*_H_ 6.82) with C-1′ (*δ*_C_ 77.0), together with the chemical shifts of C-1′, C-2′, C-6′, and C-14, also suggest the presence of a hydroxyl group at C-1′. Due to the β-orientation of CH_3_-13 and *α*-configuration of H-5 [10], the NOESY correlations of the signals H-9 (*δ*_H_ 1.79) with H-5 (*δ*_H_ 1.12, dd, *J* = 13, 2.5 Hz), H-12 (*δ*_H_ 0.86, s) with H-13 (*δ*_H_ 0.58, s), and H-13 (*δ*_H_ 0.58, s) with H-14 (*δ*_H_ 1.80, 1.89) revealed the α-configuration of H-9. Thus, penicilliumin A was elucidated as the new compound penicilliumin A and its relative stereochemistry is as shown in Figure 4. 

**Table 1:** ^13^C and ^1^H NMR data (500 MHz, CDCl_3_) for compound **1**.

Position	*δ*_C_ (m)	*δ*_H_ (m. *J* Hz)
1	38.7 CH_2_	1.05 m
		1.62 m
2	19.3 CH_2_	1.49 m
		1.52 m
3	42.0 CH_2_	1.16 m
		1.38 m
4	33.5 C	/
5	55.6 CH	1.12 dd (13, 2.5)
6	24.6 CH_2_	1.27 m
		1.74 m
7	38.1 CH_2_	1.89 m ^a^
		2.29 ddd (12.5, 6.5, 3.5)
8	148.9 C	/
9	50.5 CH	1.79 m ^b^
10	39.9 C	/
11	21.5 CH_3_	0.76 s
12	33.6 CH_3_	0.86 s
13	15.0 CH_3_	0.58 s
14	34.7 CH_2_	1.80 m ^b^
		1.89 m ^a^
15	107.0 CH_2_	4.26 br s
		4.75 br s
1′	77.0 C	/
2′	201.1 C	/
3′	134.3 CH	6.82 s
4′	150.8 C	/
5′	196.5 C	/
6′	53.0 CH_2_	2.97 d (1.6)
		3.12 d (1.6)
1′′	59.7 CH_2_	4.44 d (1.7)
		4.54 d (1.7)

^a,b^ Assignments with the same superscript in the same column may be interchanged.

The structures of known compounds **2** and **3** (Figure 4) were confirmed by detailed NMR data comparison with those in literatures [11,12]. 

### 2.3. Cytotoxic and Antiviral Activities of Penicilliumin A

So far, 7 natural analogues of penicilliumin A have been reported. Among them, tauranin [10,13] and BE-40644 [14,15] displayed potent *in vitro* cytotoxic activity against cancer cells. *In vitro* cytotoxic effects of penicilliumin A was tested against three cancer cell lines—A375, B16 and Hela—using 3-(4,5-Dimethylthiazol-2-yl)-2,5-diphenyl-tetrazolium bromide (MTT) assay. The GI_50_ values were 22.88, 27.37, and 44.05 μg/mL, respectively. In this assay, A375 cells showed relatively higher susceptibility to penicilliumin A compared to Hela and B16 cells. Penicilliumin A was also evaluated for its antiviral activity against coxsackievirus B3 (CVB3), herpes simplex virus type I (HSV-1), and influenza A virus subtype H5N3 (A/H5N3) *in vitro*. The median toxic concentration (TC_50_) values against the host cells Hela, African green monkey kidney (Vero), and Madin-Darby canine kidney (MDCK) cells were 40.72, 133.52, and 43.00 μg/mL, respectively. However, penicilliumin A did not show antiviral activity against test virus strains, under its maximal atoxic concentration (TC_0_). For further investigation of its cytotoxic activity, larger quantities of penicilliumin A are required. Optimization of fermentation conditions is currently under development by our research group to enable large scale production.

## 3. Experimental Section

### 3.1. General Experimental Procedures

MS data were acquired using a Thermo DSQ mass spectrometer. ^1^H- and ^13^C-NMR, and 2D NMR data were obtained with a Bruker Avance 500 spectrometer, with tetramethylsilane (TMS, δ 0.0 ppm) as the internal standard. The optical rotations were recorded on a 341 polarimeter. IR spectra were determined on a Nicolet 6700 FT-IR spectrometer. UV spectroscopic data were obtained on an UV-2501 PC spectrophotometer. Column chromatography (CC) was performed with silica gel (100–200 mesh; 300–400 mesh; Jiangyou Silica Gel Development, Inc., Yantai, China) and Sephadex LH-20 (Pharmacia). Thin layer chromatography (TLC, 0.1–0.2 mm or 0.3–0.4 mm) was carried out with precoated silica gel plates (GF-254, Jiangyou Silica Gel Development, Inc., Yantai, China).

### 3.2. Microbial Material

The microorganism was obtained from the sediment of the northern South China Sea (Lat. 22°6.017′N, Long. 119°17.440′E) at a depth of 1300 m. It was isolated on Gause’s No. 1 synthetic medium (soluble starch 20 g, NaCl 0.5 g, KNO_3_ 1 g, K_2_HPO_4_·3H_2_O 0.5 g, MgSO_4_·7H_2_O 0.5 g, FeSO_4_·7H_2_O 0.01 g, agar 15 g, sea water 1000 mL, pH 7.4–7.6) and designated as strain F00120. It was stored on PDA slants at 4 °C and deposited at Research Center for Marine Microbes, Chinese Academy of Sciences (Guangzhou City), as SCSIO-F00120. The colony-forming and pigmentation properties of strain F00120 were examined on Czapek’s agar (CA, consisted of sucrose 30 g, NaNO_3_ 3 g, K_2_HPO_4_ 1 g, MgSO_4_·7H_2_O 0.5 g, KCl 0.5 g, FeSO_4_ 0.01 g, NaCl 2.5 g, agar 15 g, distilled water 1000 mL), Sabourand’s agar (SA, consisted of dextrose 40 g, peptone 10 g, NaCl 2.5 g, agar 15 g, distilled water 1000 mL, pH 5.6), and potato dextrose agar (PDA, consisted of potato 200 g, dextrose 20 g, NaCl 2.5 g, agar 15 g, distilled water 1000 mL) after culturing for 7 and 14 days at 25 °C. Conidia morphology was examined of 7-day cultures grown on CA. Using the cover technique described previously [16,17], samples were observed with a Nikon Eclipse E600 light microscope and a Hitachi S-3400N scanning electron microscope. Genomic DNA isolation, PCR amplification of ITS region, sequence alignment, and phylogenetic tree construction of strain F00120 were performed as described previously [18,19].

Strain F00120 stored on PDA slants at 4 °C was cultured on PDA agar plates and incubated for 7 days. Seed medium (potato 200 g, dextrose 20 g, NaCl 2.5 g, distilled water 1000 mL) in 500-mL Erlenmeyer flasks was inoculated with strain F00120 and incubated at 25 °C for 48 h on a rotating shaker (180 rpm). Production medium of solid rice in 1000 mL flasks (rice 200 g, NaCl 0.5 g, distilled water 200 mL) was inoculated with 10 mL seed solution. Flasks were incubated at 25 °C under static stations and daylight. After 35 days, cultures from 10 flasks were harvested for the isolation of substances with cytotoxic activity.

### 3.3. Extraction and Isolation

The culture medium containing the mycelium was cut into small pieces, mixed with ethyl acetate (EtOAc) (3000 mL), and steeped for 1 day. The content was filtered under vacuum using a Buchner funnel and the extraction with EtOAc was repeated until exhaustion. The combined filtrates were washed with 5000 mL water to eliminate remaining sugar and starch. The organic phase was collected and evaporated (40 °C) to remove EtOAc. An oily residue (30.9 g) was obtained, chromatographed on silica gel and eluted with a gradient of petroleum ether (PE)–EtOAc (1:0, 30:1, 20:1, 10:1, 5:1, 1:1, 0:1) to yield 9 fractions (Frs. A1–9). Fr. A7 (5.4 g) was further separated by silica gel column chromatography and eluted with PE–acetone (4:1), resulting in 8 fractions (Frs. E1–8). Fr. E1 (976.6 mg) was chromatographed on a Sephadex LH-20 column using CHCl_3_−MeOH (1:1) to produce 6 fractions (Frs. F1–6). Then Fr. F4 (64.5 mg) was separated by Sephadex LH-20 (MeOH) to yield 3 fractions (Frs. I1–3). A further separation of Fr. I2 (41.9 mg) yielded penicilliumin A (**1**) (20 mg) by preparative thin layer chromatography using CHCl_3_–EtOAc (3:2) as developers. Fr. F4 was further separated by Sephadex LH-20 (MeOH) to yield compound **3** (10 mg). Methanol was added to the residue of Fr. A5, and the mixture was stirred to yield pure white crystal of compound **2** (213 mg).

Penicilliumin A (**1**): white crystalline solid; [α]_D_^20^: −0.008 (*c* 0.85, CHCl_3_); UV λ_max_ (cyclohexane) nm (log ε): 237 (4.22); IR (KBr) ν_max_ 3436, 1683 cm^−1^; ^1^H and ^13^C NMR data, see Table 1. HRESI-MS [M]^+^ *m/z* 360.2296 (calcd. for C_22_H_32_O_4_ 360.2295), EI-MS: *m/z* 360 [M]^+^.

### 3.4. Brine Shrimp Lethal Activity of Crude Extract of Fungal Culture and *in vitro* Cytotoxic and Antiviral Effect of Penicilliumin A

Brine shrimp lethality test for nauplii was used to determine the toxicity of EtOAc extract of fungal culture as in the report of Solis *et al.* [20]. Cytotoxic effect of penicilliumin A was assayed against 3 tumor cell lines, including B16, A375, and Hela, according to previously described methods [19]. Antiviral activity against 3 virus strains CVB3, HSV-1, and A/H5N3 of this compound was evaluated by the cytopathic effect (CPE) inhibitory assay as described previously [21,22]. Cell lines Hela, Vero and MDCK were used as host cells of CVB3, HSV-1, and A/H5N3, respectively.

## 4. Conclusions

A fungal strain, *Penicillium* sp. F00120, was isolated from a deep sea sediment sample during a screening program and the EtOAc extract displayed strong toxic activity against brine shrimp larvae. The identity of the isolate was determined by morphological and genetic analyses. One new sesquiterpene quinone penicilliumin A (**1**), and two known compounds ergosterol (**2**) and ergosterol peroxide (**3**), were isolated from the culture of solid-state fermentation of this strain. Penicilliumin A exhibited *in vitro* cytotoxic activity against B16, A375, and Hela cells.
